# Consider this a WARNing

**DOI:** 10.1016/j.patter.2024.101009

**Published:** 2024-06-14

**Authors:** Sam Freesun Friedman, Shaan Khurshid

**Affiliations:** 1 Data Sciences Platform, Broad Institute of MIT and Harvard, Cambridge, MA, USA; 2 Cardiology Division, Massachusetts General Hospital, Boston, MA, USA; 3 Cardiovascular Disease Initiative, Broad Institute of MIT and Harvard, Cambridge, MA, USA; 4 Cardiovascular Research Center, Massachusetts General Hospital, Boston, MA, USA

## Abstract

Atrial fibrillation (AF) prediction can be valuable at many timescales and in many populations. In this issue of *Patterns*, Gavidia et al. train a model called WARN for short-term prediction of AF in the timescale of minutes in patients wearing 24-h continuous Holter electrocardiograms. The ability to predict near-term (e.g., 30 min) AF has the potential to enable preventive therapies with rapid mechanisms of action (e.g., oral anticoagulation, anti-arrhythmic drugs). In this way, efficient, continuous, and algorithmic monitoring of AF risk could reduce burden on healthcare workers and represents a valuable clinical pursuit.

## Main text

Atrial fibrillation (AF) is the most common abnormal heart rhythm and is associated with significant health risks as well as substantial healthcare utilization related to acute care visits and increased hospitalizations. In recent years, deep learning has emerged as a powerful tool in processing electrocardiograms (ECGs) for tasks like classifying arrhythmias,^1^ detecting the presence of AF,^2^^,^^3^ and even predicting the future incidence of AF.^4^^,^^5^ Prior work focused on AF prediction has generally considered timescales of years for primary care patients with longitudinal follow up, with the intent of identifying at-risk individuals who may benefit from longer-term preventive efforts (e.g., weight loss, alcohol cessation, blood pressure control) with potential to reduce risk of AF-related complications or perhaps even prevent AF altogether.^6^

But AF prediction can be valuable at many timescales and in many populations. In this issue of *Patterns*, Gavidia et al.^7^ train a model for short-term prediction of AF in the timescale of minutes in patients wearing 24-h continuous Holter ECGs. The ability to predict near-term (e.g., 30 min) AF has the potential to enable preventive therapies with rapid mechanisms of action (e.g., oral anticoagulation, anti-arrhythmic drugs). In this way, efficient, continuous, and algorithmic monitoring of AF risk could reduce the burden on healthcare workers and represent a valuable clinical pursuit.

This study develops WARN (warning of atrial fibrillation), a deep convolutional neural network (CNN) that processes R-to-R interval (RRI) signals, which are lossy 2D encodings of the raw 1D waveform coming from the continuous single-lead Holter ECG. While a focus on RRIs rather than the complete ECG waveform does come with some performance penalties, it also leads to massive efficiency gains, allowing for real-time model inference with minimal computing costs. This proactive approach offers the potential for timely interventions leveraging continuous monitoring. Model development included recordings from 280 patients used for training and validation, with records from an additional 70 patients used for testing. WARN’s robustness was further assessed using data from 33 patients across two external centers; importantly, those centers represented varying geographic locations and patient populations, allowing us to glean insight into how the model generalizes.

Indeed, the performance in the external centers is quite different from the held-out test set. As is often observed,^8^ there was a performance drop in the external centers with the area under the reciever operating characteristic curve going from 0.9 to 0.8 and the area under the precision-recall curve decreasing from 0.88 to 0.73. The drop indicates there is some learning on the development cohort in Tongji Hospital in China that appears not to generalize to the cohorts in Argentina or France. Additionally, the external datasets contained just 33 people, whereas cohorts of thousands of people or more are needed for more robust estimates of generalizability. The derivation set of only 350 patients is also small compared to the tens of thousands of individuals being phenotyped in many modern biobanks and clinical cohorts (where continuous 24-h ECG recordings are admittedly not yet readily available).^9^ That being said, WARN does achieve an impressive accuracy of 83% and an F1-score of 85% on the held-out test data. Furthermore, despite lower accuracy in the external cohorts, WARN consistently demonstrated the ability to predict an incipient AF episode approximately 30 min before onset. These results underscore the model’s potential for short-term AF prediction, providing a reasonable lead time for potential preventive measures. The authors gesture to the potential of applying models like WARN to wearable technology (e.g., smartwatches), where WARN may offer a scalable solution for continuous AF risk monitoring, accessible to a broad patient population. The low computational cost should offer feasibility for real-time applications, making it a promising tool for personalized healthcare.

The integration of models like WARN into clinical practice could lead to significant advancements in AF management. Early warning systems offer the potential for proactive, rather than reactive, healthcare, which may reduce the burden on resource-intensive emergency services and other acute care settings. There are several important barriers that must be addressed before successful clinical deployment, however. First, studies including larger samples representing more diverse clinical populations are needed to ensure discrimination is consistent, particularly given evidence of generalization cost in the current analysis. Second, prospective assessments are needed to assess whether accuracy is sufficient to guide effective clinical decisions, particularly given the fact that the implications of false positives can be substantial for certain therapies with potential for on-demand use (e.g., oral anticoagulation, anti-arrhythmic drugs). Third, despite recent interest, the effectiveness of “on-demand” therapies for incipient AF is unproven, with evidence limited to small pilot studies.^10^ For certain potential applications (e.g., use of an anti-arrhythmic drug to “avert” an AF episode), even 30 min might not be enough time for the medications to take effect. Fourth, although a focus on RRIs as a simpler and potentially more general metric offering lower computational cost lends itself well to use with wearable and mobile devices, it is important to recognize that real-world tracings from such devices are subject to limitations that may nevertheless impact performance (e.g., acquisition noise, data interruptions).

Despite these open questions, algorithms such as WARN offer insight into how we may utilize individual risk prediction to enable more effective (and acute) risk-mitigation strategies for important conditions such as AF. Even beyond the clinic, the WARN algorithm may also find use as a tool for biological discovery. Researchers have shown that ECG-derived model predictions can help elucidate the genetic architecture of arrhythmias and related conditions.^11^^,^^12^ In the future, visits to the cardiologist may entail a suite of AI-derived phenotypes being assessed on raw patient data like the ECG using models like WARN. Here, AI could serve to both personalize care for the patient at hand while globalizing insights across patients, cohorts, and healthcare centers as each new datapoint contributes to training the next generation of models.
